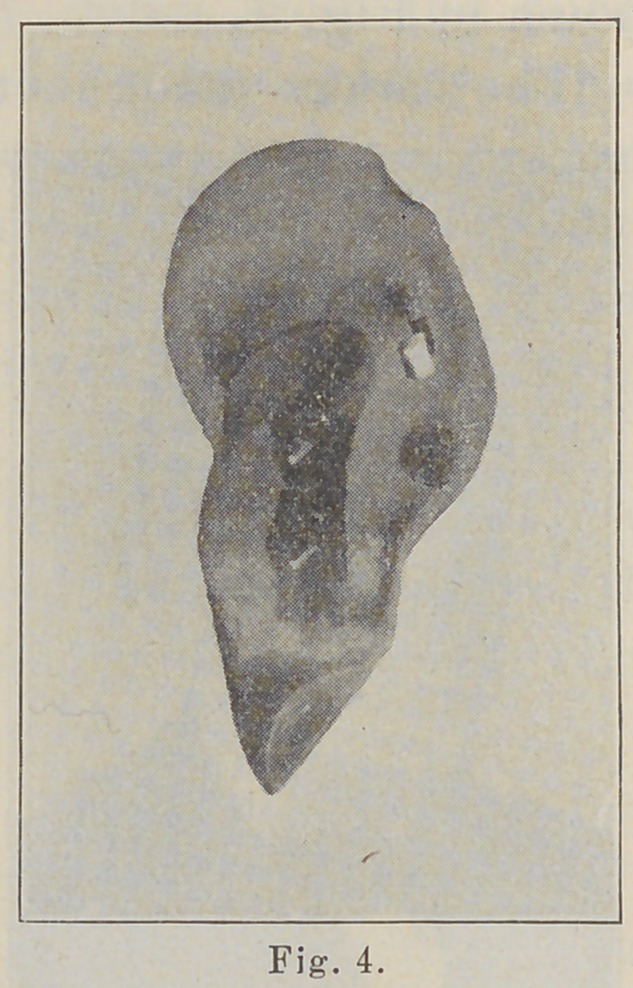# A Facial Restoration with Vulcanite

**Published:** 1901-05-15

**Authors:** J. A. Heidbrink


### A FACIAL RESTORATION WITH VULCANITE.

BY J. A. HEIDBRINK.

The following case is reported from the prosthetic clinic of the dental college of the University of Michigan. The patient had been treated for cancer of the superior maxilla by a country physician who used chemical cauterants and destroyed a large portion of the jaw and overlying tissues. The condition is fairly well shown in Fig. 1.

The cancer made its appearance in the form of a

Fig. 1.

Fig. 2.

thickening and whitening of the mucous membrane of the palate. As it developed it involved the left maxillary bone and the tissues of the left cheek to some extent. The operation was performed by chemical cautery, and the entire left superior maxilla removed, together with the surrounding soft tissues. An effort was made to preserve sufficient tissue to form the upper lip and the cheek, which perhaps would have been accomplished had not the patient unfortunately torn it away during a fit of insanity brought about by the severe pain attending the operation. The entire cavity of the mouth was thus exposed, and an opening made into the inferior meatus of the nose, showing the inferior terbinated bone. The skin and scar tissue attached itself to the bones, forming the superior margin of the orifice in such a way that the border served an excellent purpose for support for the plate that was fitted to the case. The wound was perfectly healed and the tissues not

Fig. 3.

Fig. 4.

very tender. All the teeth from the first bicuspid on the right side to the first molar on the left, inclusive, were in place below; and those from the right central incisor to the first bicuspid above. The patient, as is very evident, had great difficulty in speech and the taking of food. In the accomplishment of either it was necessary for him to fill in the cavity with gauze. A special tray was made and adapted to the case and a modeling compound impression taken. The opening into the nasal fossa was first packed with cotton.

Special care was taken to get a sharp impression of the superior and posterior borders which were to be used for the support of the left side of the plate. A rubber plate was then made, restoring the palate and completing the superior arch. Fig. 2 shows the plate in place.

How to restore the cheek was a troublesome problem for some time. It was finally decided to make a shell of hard rubber vulcanite. Flesh colored rubber was procured. The shell was vulcanized in two pieces and made to conform to the lower lip, the vertical margin of the upper lip, and the gum margin of the plate, the superior border overlapping the edge of the plate. The shell was allowed to extend somewhat over the margin at the angle of the orifice to fill out a depression caused by the contraction of the scar tissue. Thus the upper lip and cheek were restored as shown in Fig. 3.

The shell was made detachable to facilitate cleansing. The inside of shell is shown in Fig. 4. The pins pass into corresponding holes in the plate, and the spring clasp passes back of a rim of rubber, extending transversely across the upper border of the plate. See Fig 2.

The appliance, while not as satisfactory as might be desired, adds as much to the comfort of the patient as can be expected under the circumstances. In appearance he is much more presentable than before. It incloses the oral cavity ; restores the hard palate and the upper arch. This makes possible much more distinctness in speech and immensely greater ease and comfort in mastication.

## Figures and Tables

**Fig. 1. f1:**
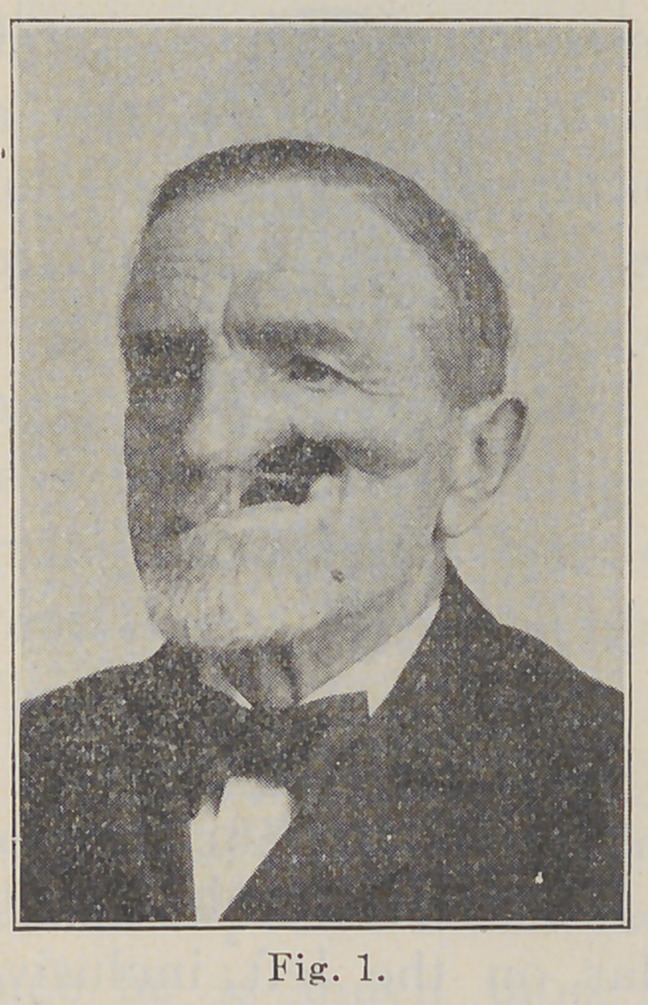


**Fig. 2. f2:**
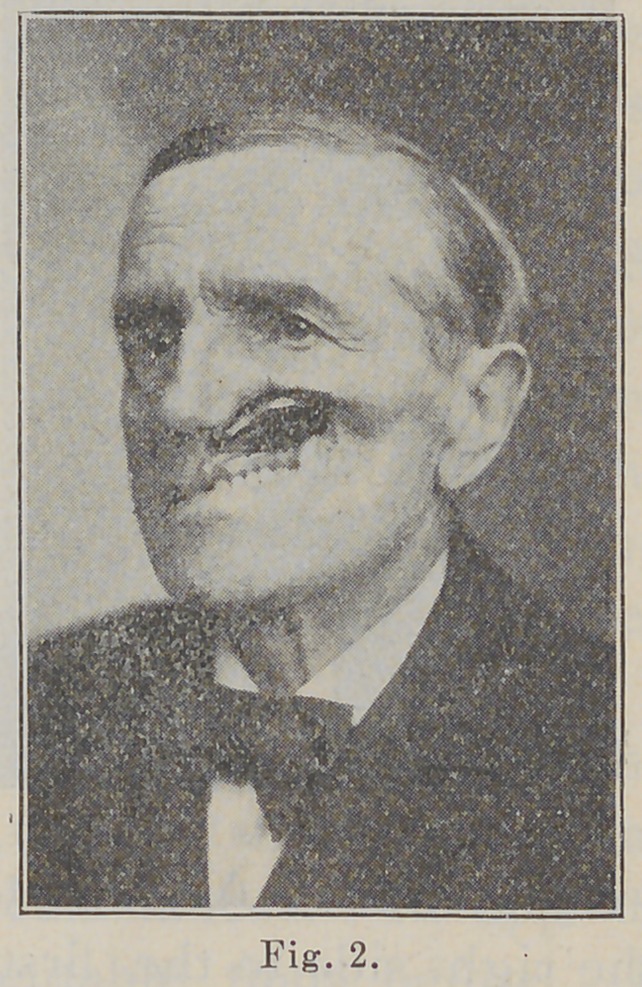


**Fig. 3. f3:**
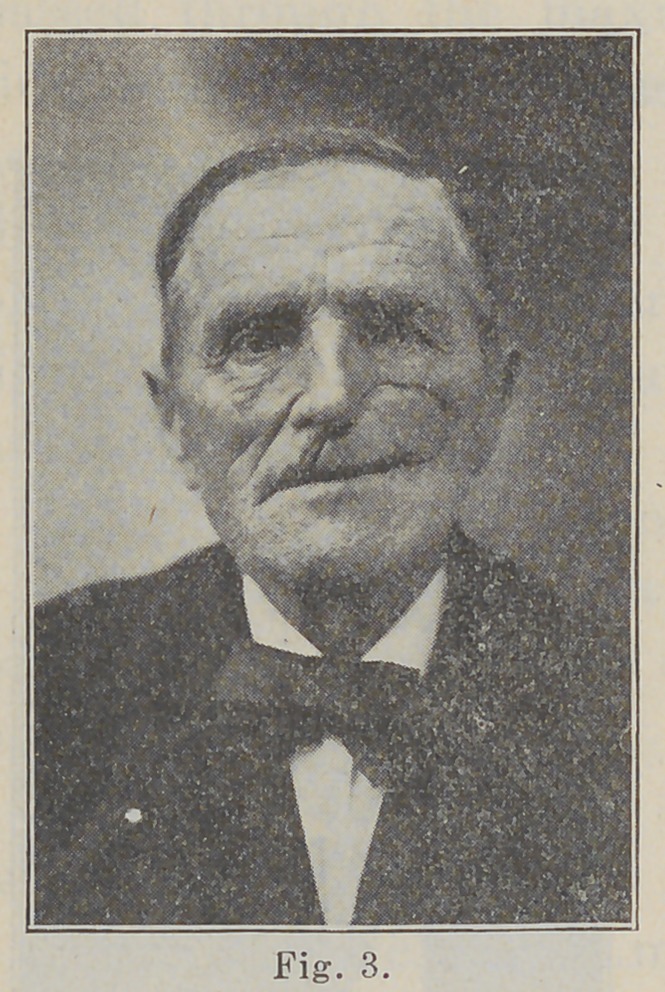


**Fig. 4. f4:**